# O Papel do Volume Indexado do Átrio Esquerdo na Detecção de Desfechos Desfavoráveis em Pacientes com Doença Pulmonar Obstrutiva Crônica: Estudo de Coorte

**DOI:** 10.36660/abc.20250131

**Published:** 2025-11-19

**Authors:** Maria Clara C Sposito, Leticia A. Branco, Jaqueline Bianchi, Fernanda O. Duarte, Krissia F. Godoy, Joice M. A. Rodolpho, Bruna Dias de Lima Fragelli, Renan S. Marinho, Stella Maris Firmino, Audrey Borghi Silva, Fernanda Freitas Anibal, Meliza Goi Roscani

**Affiliations:** 1 Universidade Federal de São Carlos São Carlos SP Brasil Universidade Federal de São Carlos, São Carlos, SP – Brasil

**Keywords:** Enfisema Pulmonar, Prognóstico, Biomarcadores

## Abstract

**Fundamento:**

A doença pulmonar obstrutiva crônica (DPOC) é uma condição progressiva com alta mortalidade. A compreensão dos preditores de desfechos desfavoráveis pode auxiliar no desenvolvimento de abordagens terapêuticas individualizadas e na tomada de decisões sobre internações hospitalares ou em unidades de terapia intensiva.

**Objetivo:**

Avaliar o perfil epidemiológico e laboratorial de pacientes com DPOC e correlacionar variáveis clínicas, ecocardiográficas e de biomarcadores com desfechos desfavoráveis por exacerbação.

**Métodos:**

Trata-se de um estudo prospectivo envolvendo pacientes com DPOC submetidos a avaliação clínica, espirometria, ecocardiografia transtorácica e exames laboratoriais. Os pacientes foram acompanhados durante 1 ano para monitoramento de desfechos desfavoráveis. O nível de significância considerado para todas as análises foi de p < 0,05.

**Resultados:**

Foram incluídos 228 pacientes, com média de idade de 71 ± 9 anos. A maioria dos pacientes era do sexo masculino (58%), com predomínio de tabagismo (72%), hipertensão arterial (66%) e GOLD B (55%). O volume indexado do átrio esquerdo (ViAE) demonstrou boa acurácia na detecção de hospitalização por exacerbação, com ponto de corte de 36,5 ml/m^2^, apresentando sensibilidade de 100% e especificidade de 70%. Pacientes com ViAE > 36,5 ml/m^2^ apresentaram piora da função diastólica e sistólica, conforme indicado pela velocidade de pico da onda E mitral na fase de enchimento rápido/velocidade E′ de deslocamento do anel mitral septal ou lateral na fase de enchimento rápido (E/E′) (p = 0,02) e fração de ejeção do ventrículo esquerdo (p = 0,01), juntamente com níveis elevados de fator de necrose tumoral alfa (p = 0,05) e NT-proBNP (p = 0,03).

**Conclusão:**

O ViAE pode ser um marcador confiável para a predição de desfechos desfavoráveis em pacientes com DPOC. Estratégias individualizadas devem ser implementadas para melhorar a gravidade da doença e a função cardiovascular.

## Graphical abstract

**Figura Central:**
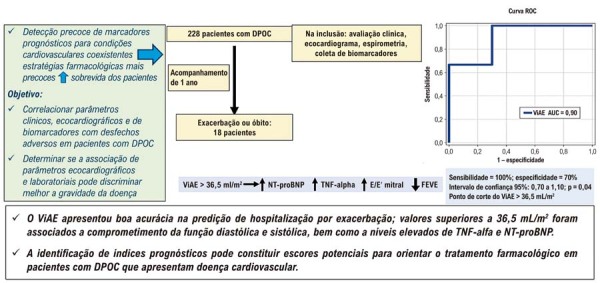
O Papel do Volume Indexado do Átrio Esquerdo na Detecção de Desfechos Desfavoráveis em Pacientes com Doença Pulmonar Obstrutiva Crônica: Estudo de Coorte

AUC: área sob a curva; DPOC: doença pulmonar obstrutiva crônica; FEVE: fração de ejeção do ventrículo esquerdo; ROC: característica operacional do receptor; TNF: fator de necrose tumoral; ViAE: volume indexado do átrio esquerdo.

## Introdução

A doença pulmonar obstrutiva crônica (DPOC) é uma condição progressiva associada a alta morbimortalidade, representando um desafio significativo para a saúde pública. Marcadores prognósticos bem estabelecidos, como idade, volume expiratório forçado no primeiro segundo (VEF_1_), índice de massa corporal e tabagismo, podem predizer desfechos desfavoráveis, incluindo morte, risco de exacerbação aguda e readmissão hospitalar em pacientes com DPOC.^1 , 2^ Estudos indicaram a pró-adrenomedulina e o teste de caminhada de 6 minutos como preditores independentes de mortalidade em 2 anos para pacientes com DPOC. Adicionalmente, outros estudos destacaram a importância de biomarcadores como D-dímero, troponina, peptídeo natriurético tipo B (BNP), interleucina 6 (IL-6), interleucina 8 (IL-8) e proteína C reativa (PCR) como preditores significativos de desfechos adversos na DPOC.^3^

A coexistência de insuficiência cardíaca em pacientes com DPOC é bem reconhecida, visto que essas condições compartilham vários fatores de risco comuns, incluindo tabagismo, inflamação sistêmica e idade avançada. Pesquisas sugerem que níveis elevados de PCR e BNP, secretados por cardiomiócitos danificados, são prevalentes na DPOC. Ademais, estudos têm destacado a importância do D-dímero, troponina, IL-6 e IL-8 como importantes preditores de desfechos desfavoráveis nesses pacientes.^4 - 6^ A troponina, conforme observado em vários estudos, pode estar elevada em pacientes hospitalizados por exacerbações da DPOC.^7^

Dada a associação de comorbidades cardiovasculares em pacientes com DPOC, acredita-se que a ecocardiografia possa desempenhar um papel crucial na detecção de sinais de gravidade da doença.^8^ Cassagnes et al. constataram que o volume indexado do átrio esquerdo (ViAE) foi um marcador de evolução desfavorável em pacientes com DPOC. No entanto, outro estudo não relatou diferença na deformação atrial esquerda entre pacientes com DPOC e indivíduos saudáveis.^9^ A relação entre variáveis ecocardiográficas e biomarcadores prognósticos na predição de desfechos desfavoráveis na DPOC permanece inadequadamente elucidada.^10^ Rawy et al. observaram uma alta prevalência de disfunção diastólica do ventrículo esquerdo (VE) em pacientes com DPOC, que foi associada ao aumento da gravidade da doença e a níveis elevados de marcadores inflamatórios, independentemente de outros biomarcadores, como os níveis de PCR.^10 , 11^

Considerando esses achados, a quantificação, o registro, o monitoramento e a análise dessas variáveis e suas associações podem desempenhar um papel importante na definição do prognóstico e na identificação de pacientes com maior gravidade e progressão desfavorável da doença. Além disso, a detecção precoce de marcadores prognósticos para condições cardiovasculares coexistentes pode facilitar estratégias farmacológicas mais precoces, potencialmente alterando o curso da doença e melhorando a sobrevida dos pacientes.

Portanto, o objetivo do presente estudo foi correlacionar dados clínicos, ecocardiográficos e de biomarcadores com desfechos desfavoráveis em pacientes com DPOC e determinar se a associação de parâmetros ecocardiográficos e laboratoriais pode discriminar melhor a gravidade da doença.

## Métodos

Foi realizado um estudo de coorte para acompanhar pacientes com diagnóstico confirmado de DPOC atendidos em um ambulatório de pneumologia. O projeto foi aprovado pela Comissão Nacional de Ética em Pesquisa e pelos Comitês de Ética em Pesquisa dos Centros de Saúde (CAAE 63414822.1.0000.5504).

O presente estudo foi realizado no período de janeiro de 2022 a outubro de 2023 e seguiu as diretrizes STROBE (Fortalecimento do Relato de Estudos Observacionais em Epidemiologia).

A seleção dos pacientes foi baseada na avaliação de prontuários eletrônicos de consultas ambulatoriais.

Os critérios de inclusão foram: obtenção do consentimento informado; idade superior a 40 anos; diagnóstico confirmado de DPOC, definido como espirometria pós-broncodilatador mostrando VEF_1_/capacidade vital forçada (CVF) < 70%; e ausência de limitações físicas.

Os critérios de exclusão foram: pacientes com limitações biomecânicas ou psiquiátricas que impedissem o preenchimento de questionários ou a realização de exames; e pacientes com exacerbação da DPOC nos 3 meses anteriores à inclusão.

No momento da inclusão, todos os pacientes foram submetidos a avaliação clínica, ecocardiograma transtorácico realizado por um cardiologista com vasta experiência e espirometria pré e pós-broncodilatador realizada por um pneumologista treinado. Adicionalmente, foram coletados exames laboratoriais de sangue. Todos os pacientes incluídos foram monitorados por 1 ano para investigar os desfechos primários, que incluíram hospitalização por exacerbação da doença (caracterizada por aumento da dispneia e/ou tosse e piora da expectoração em 14 dias, acompanhada de taquipneia ou taquicardia) e mortalidade.^12^ Os dados sobre os desfechos primários foram obtidos por meio de prontuários eletrônicos e ligações telefônicas de acompanhamento para os pacientes ou seus familiares.

### Avaliação clínica

Os pacientes foram avaliados quanto a sintomas como tosse, produção de escarro, coloração do escarro, falta de ar, tolerância ao exercício, hospitalização prévia por exacerbação, tabagismo, uso de oxigenoterapia domiciliar, medicamentos em uso e presença de comorbidades, incluindo hipertensão arterial sistêmica, diabetes mellitus, insuficiência cardíaca, doença arterial coronariana, doença renal crônica e obesidade. A escala de dispneia modificada do Medical Research Council (mMRC) e o teste de avaliação da DPOC (CAT) também foram utilizados durante as avaliações clínicas. O exame físico completo incluiu medidas de pressão arterial sistêmica, frequência respiratória, frequência cardíaca, saturação de oxigênio, índice de massa corporal e presença de estertores pulmonares.^13 , 14^

### Ecocardiografia Doppler

Para a estratificação clínica e diagnóstica, os pacientes com DPOC foram submetidos a um ecocardiograma bidimensional utilizando um aparelho de ultrassom “Affiniti 50” (Philips, EUA) com transdutor de 2–4 MHz e software de imagem Doppler tecidual. Um único cardiologista realizou todas as avaliações. A quantificação das câmaras cardíacas foi realizada de acordo com as diretrizes da Sociedade Americana de Ecocardiografia.^15^ Foram medidos o diâmetro diastólico do VE, o ViAE (pelo método de Simpson), a massa indexada do VE, a espessura relativa da parede e o diâmetro diastólico basal do ventrículo direito. As dimensões do ventrículo direito foram estimadas seguindo diretrizes recentes, utilizando a visão apical de 4 câmaras focada no ventrículo direito. O diâmetro diastólico do ventrículo direito foi definido como a dimensão transversal máxima no terço basal do trato de entrada do ventrículo direito no final da diástole. Os padrões de entrada mitral e tricúspide foram avaliados medindo as velocidades de enchimento diastólico precoce (E) e tardio (A) e calculando a razão E/A. As velocidades longitudinais dos ventrículos esquerdo e direito nos respectivos níveis anulares mitral e tricúspide foram obtidas usando Doppler tecidual de onda pulsada guiado por cores em visão apical de 4 câmaras. As medições do Doppler espectral incluíram a velocidade longitudinal de pico sistólico (s′) e a velocidade de enchimento diastólica inicial (e′), calculadas em média ao longo de 5 ciclos cardíacos consecutivos. A razão E/e′ foi calculada para avaliar a função diastólica, enquanto a fração de ejeção do ventrículo esquerdo (FEVE) foi derivada pelo método de Teicholz, dependendo da ausência de anormalidades regionais do movimento da parede. A função sistólica do ventrículo direito foi avaliada por meio da análise subjetiva do movimento da parede, excursão sistólica do plano anular tricúspide e pico de velocidade sistólica do anel tricúspide.

### Espirometria

Foram obtidas espirometrias pré e pós-broncodilatador (Masterscreen Body, Mijnhardt/Jäger, Würzburg, Alemanha), que forneceram medidas de VEF_1_ e CVF, permitindo o cálculo da razão VEF_1_/CVF. A espirometria foi realizada de acordo com as recomendações das diretrizes da Sociedade Torácica Americana/Sociedade Respiratória Europeia.^16^ A gravidade da limitação do fluxo aéreo na DPOC foi classificada com base nos critérios da Iniciativa Global para Doença Pulmonar Obstrutiva Crônica (GOLD), categorizando os pacientes como portadores de doença moderada (GOLD II), grave (GOLD III) ou muito grave (GOLD IV).^12^

### Avaliações dos sintomas

Os sintomas dos pacientes foram avaliados após 12 meses de acompanhamento utilizando a escala de dispneia mMRC, que mede a gravidade da dispneia apresentada pelo paciente. Além disso, o CAT foi utilizado para avaliar o impacto dos sintomas em pacientes com DPOC; no entanto, o CAT não classifica os pacientes em categorias de gravidade. No CAT, as pontuações superiores a 25 são pouco frequentes em pacientes saudáveis, enquanto pontuações abaixo de 25 são incomuns em pacientes com DPOC.^13 , 14^

### Biomarcadores

Foram coletados os seguintes exames laboratoriais de sangue na inclusão: D-dímero, NT-proBNP, cortisol, PCR, PCR de alta sensibilidade, IL-6, IL-8 e fator de necrose tumoral (TNF) alfa.

### Procedimentos de acompanhamento

Os pacientes foram incluídos ao longo de um período de 12 meses por meio de ligações telefônicas e consultas médicas de rotina com seus cuidadores familiares. As investigações se concentraram em desfechos desfavoráveis, com prontuários médicos revisados durante as ligações telefônicas e as consultas.

### Análise estatística

Todas as análises foram realizadas no Sigma Plot Systat, versão 12.0. Os resultados foram relatados como média e desvio-padrão para variáveis contínuas com distribuição normal, como mediana e intervalo interquartil para variáveis contínuas sem distribuição normal e como frequências e porcentagem para variáveis categóricas. O teste t de Student não pareado foi aplicado para comparações de variáveis contínuas, enquanto o teste U de Mann Whitney foi utilizado para variáveis ordinais. O teste de Shapiro-Wilk foi realizado para verificar a normalidade dos dados. O teste qui-quadrado foi empregado para avaliar a independência para variáveis categóricas. Regressão linear múltipla foi realizada. Uma análise de regressão linear múltipla foi realizada para predizer os valores das variáveis dependentes. Por fim, foram construídas curvas de característica operacional do receptor (ROC) para variáveis contínuas e ordinais referentes a desfechos como óbito, exacerbação, hospitalização, atendimentos de emergência e desfechos negativos, com a área sob a curva (AUC) calculada para avaliar o desempenho diagnóstico. O nível de significância considerado para todas as análises foi de p < 0,05

## Resultados

Foram incluídos 228 pacientes no estudo. As características clínicas e laboratoriais basais são apresentadas na Tabela 1 . A média de idade foi de 71 ± 9 anos, com notável prevalência do sexo masculino e hipertensão arterial sistêmica. A maioria dos pacientes foi classificada no grupo GOLD B. Os desfechos primários foram observados em aproximadamente 7,9% da coorte. A avaliação ecocardiográfica revelou FEVE preservada de 63,9% ± 7,5%. No entanto, houve um aumento na média do ViAE de 42,7 ± 28,2 ml/m^2^, juntamente com a pressão sistólica da artéria pulmonar estimada de 46,8 ± 15,4 mmHg. Adicionalmente, os níveis de NT-proBNP estavam elevados no momento da inclusão.

**Tabela 1:** – Características clínicas e laboratoriais basais de pacientes com DPOC

Variáveis	N = 228
Idade, anos	71 ± 9
Sexo masculino, n (%)	132 (57,9)
Tabagismo ativo, n (%)	62 (27,1)
Ex-tabagismo, n (%)	150 (65,8)
Tempo de tabagismo, anos	37,2 ± 18,3
Histórico tabágico, anos-maço	63,2 ± 55,2
Tempo sem fumar, anos	10,3 ± 12,7
**Comorbidades**	
Asma, n (%)	30 (13,2)
Diabetes mellitus tipo 2, n (%)	32 (14,0)
HAS, n (%)	151 (66,3)
DAC, n (%)	4 (1,8)
Obesidade, n (%)	30 (13,1)
IMC (kg/m^2^)	25,6 ± 8,0
**Escala de gravidade dos sintomas**	
mMRC (0 a 5)	3,0 [1,0 a 3,0]
Escore CAT (0 a 40)	19,0 [9,0 a 25,0]
**Espirometria**	
CVF, L	2,5 ± 1,0
CVF, %	74,2 ± 24,1
VEF_1_, L	1,5 ± 0,7
VEF_1_, %	56,1 ± 21,4
VEF_1_/CVF	0,6 ± 0,2
VEF_1_/CVF, %	18,3 ± 37,5
VEF_1_/CVF pós-broncodilatador, L	0,7 ± 0,1
VEF_1_/CVF pós-broncodilatador, %	50,0 ± 48,3
**Classificação GOLD e ABE**	
**GOLD**	2,0 [2,0 a 3,0]
**Classificação ABE**	
A	24,0%
B	56,0%
E	20,0%
**Ecocardiograma**	
DDVE, mm	47,7 ± 10,0
DDVD basal, mm	33,9 ± 9,4
Espessura relativa da parede	0,4 ± 0,1
ViAE, ml/m^2^	39,92 ± 19,33
MiVE, g/m^2^	99,6 ± 33,3
ViAD, ml/m^2^	38,3 ± 46,2
E mitral, cm/s	66,5 ± 20,8
S′ mitral, cm/s	9,1 ± 2,2
E′ do anel septal mitral, cm/s	7,5 ± 2,7
E′ do anel lateral mitral, cm/s	8,8 ± 3,1
Razão E/E′ mitral	10,2 ± 4,0
E tricúspide, cm/s	44,0 ± 13,1
Pressão sistólica da artéria pulmonar, mmHg	46,8 ± 15,4
TAPSE, mm	20,9 ± 6,1
FEVE, %	63,9 ± 7,5
Deformação longitudinal global do VE, %	−12,2 ± 14,5
**Biomarcadores**	
D-dímero, mg/L	0,7 ± 0,8
Mioglobina, ng/mL	17,6 ± 14,8
NT-proBNP, pg/mL	330,0 [234,2 a 599,0]
PCR, mg/L	6,8 ± 6,9
PCR de alta sensibilidade, mg/L	4,6 ± 6,6
IL-1, pg/mL	6,8 ± 5,8
IL-6, pg/mL	6,9 ± 5,4
IL-8, pg/mL	13,2 ± 10,4
TNF alfa, pg/mL	15,0 ± 22,9
**Medicações, n (%)**	
SABA	96 (42,1)
LABA + CI	54 (23,4)
LAMA	44 (19,3)
LABA + LAMA	3 (1,3)
Desfechos primários, n (%)	18 (7,9)
Óbito	4 (1,8)
Hospitalização por exacerbação	14 (6,1)

Valores apresentados como média ± desvio padrão para variáveis com distribuição normal ou mediana e intervalo interquartil para variáveis não paramétricas ou número (N) e porcentagem (%). Valores de referência aplicados – D-dímero: 0 a 0,5 mg/L; CK-MB: 0 a 5 ng/ml; NT-proBNP: 0 a 300 pg/mL < 75 anos e 0 a 450 ≥ 75 anos; PCR: 0 a 10 mg/L; PCR de alta sensibilidade: 0 a 1 mg/L; mioglobina: 0 a 58 ng/mL; IL-6: < 5,9 pg/ml; IL-1: < 5 pg/mL; TNF-alfa: < 8,1 pg/mL; IL-8: < 62 pg/mL. Classificação GOLD ABE – A: não exacerbadores, sintomas baixos; B: não exacerbadores, sintomas altos; E: exacerbadores. CAT: teste de avaliação da DPOC; CI: corticoides inalatórios; CVF: capacidade vital forçada; DAC: doença arterial coronariana; DDVD: diâmetro diastólico do ventrículo direito; DDVE: diâmetro diastólico do ventrículo esquerdo; E: velocidade de pico da onda E na fase de enchimento rápido mitral ou tricúspide; E′ mitral: velocidade de deslocamento do anel mitral septal ou lateral na fase de enchimento rápido; E′ tricúspide: velocidade de deslocamento do anel tricúspide lateral na fase de enchimento rápido; FEVE: fração de ejeção do ventrículo esquerdo; GOLD: Iniciativa Global para Doença Pulmonar Obstrutiva Crônica; IMC: índice de massa corporal; LABA: beta agonista de longa ação; LAMA: antagonista muscarínico de longa ação; MiVE: massa indexada do ventrículo esquerdo; mMRC: escala de dispneia modificada do Medical Research Council; NT-proBNP: fragmento N-terminal do peptídeo natriurético tipo B; PCR: proteína C-reativa; SABA: beta agonista de curta ação; SAMA: antagonista muscarínico de curta ação; TAPSE: excursão sistólica do plano anular tricúspide; TNF: fator de necrose tumoral; VEF1: volume expiratório forçado no primeiro segundo; ViAD: volume indexado do átrio direito; ViAE: volume indexado do átrio esquerdo.

Pacientes que apresentaram desfechos desfavoráveis, incluindo óbito e/ou hospitalização por exacerbação, apresentaram sintomas piores e função pulmonar mais comprometida no momento da inclusão, conforme mostrado na Tabela 2 .

**Tabela 2:** – Comparação de pacientes com DPOC em relação aos desfechos primários ao longo de um período de acompanhamento de 1 ano

Variáveis	Desfechos primários, sim (N = 18)	Desfechos primários, não (N = 210)	Valor p
Escore CAT	26 [15 a 31]	17 [7 a 20]	**0,032**
Limitação nas atividades (0 a 5)	5,0 [3,3 a 5,0]	0,0 [0,0 a 3,0]	**0,019**
Confiança para sair de casa (0 a 5)	1,0 [0,0 a 5,0]	0,0 [0,0 a 0,0]	**0,041**
GOLD	3,0 [2,0 a 3,5]	2,0 [2,0 a 3,0]	**0,039**
CVF, %	53,3 ± 14,7	69,2 ± 18,2	**0,02**
VEF_1_, %	43,2 ± 17,4	59,0 ± 17,7	**0,025**
VEF_1_ pós-broncodilatador, %	46,1 ± 17,7	60,4 ± 16,8	0,063

Valores apresentados como média ± desvio-padrão para variáveis com distribuição normal ou mediana e intervalo interquartil para variáveis não paramétricas. Comparação baseada no teste t ou Mann-Whitney para média e mediana, respectivamente; nível de significância p < 0,05. CVF: capacidade vital forçada; DPOC: doença pulmonar obstrutiva crônica; VEF1: volume expiratório forçado no primeiro segundo.

**Figura 1:**
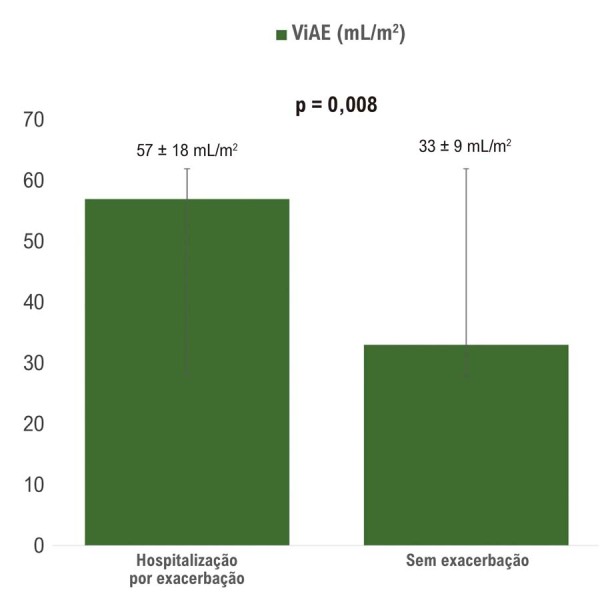
– Comparação do ViAE (mL/m2) entre pacientes com e sem hospitalização por exacerbação da DPOC. DPOC: doença pulmonar obstrutiva crônica; ViAE: volume indexado do átrio esquerdo.

Em uma análise isolada, os pacientes que necessitaram de hospitalização por exacerbação ao longo do período de 1 ano apresentaram um aumento significativo na ViAE (p = 0,008), conforme mostrado na Figura 1 . Em uma regressão linear múltipla ajustada para o escore CAT, apenas NT-proBNP (p = 0,03) e PCR (p = 0,03) foram significativamente associados ao aumento do ViAE (R = 0,83; p = 0,006).

**Figura 2:**
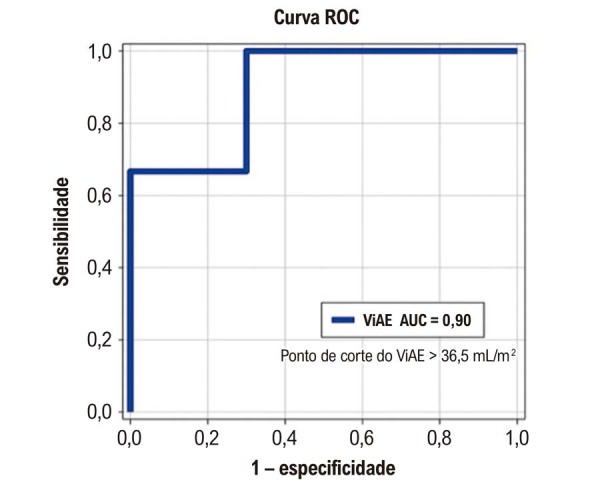
– A análise da curva ROC mostra uma AUC de 0,90 (intervalo de confiança de 95%: 0,70 a 1,10; p = 0,04), com sensibilidade de 100% e especificidade de 70%. Foi identificado um ponto de corte ideal do ViAE de > 36,5 mL/m2 para predizer hospitalização por exacerbações em pacientes com DPOC durante o período de acompanhamento de 1 ano. AUC: área sob a curva; DPOC: doença pulmonar obstrutiva crônica; ROC: característica operacional do receptor; ViAE: volume indexado do átrio esquerdo.

Considerando as variáveis clínicas e ultrassonográficas, o ViAE demonstrou boa acurácia na predição de hospitalização por exacerbação. A curva ROC indicou uma AUC de 0,90 (intervalo de confiança de 95%: 0,70 a 1,10; p = 0,04) com ponto de corte de > 36,5 ml/m^2^. Esse limiar apresentou sensibilidade de 100% e especificidade de 70% para prever esse desfecho adverso. Esses resultados são apresentados na Figura 2 .

Aplicando o ponto de corte identificado na análise ROC, comparamos as variáveis para valores de ViAE abaixo ou acima de 36,5 ml/m^2^, conforme apresentado na Tabela 3 . Pacientes com ViAE > 36,5 ml/m^2^ apresentaram piora da função diastólica e sistólica, indicada pela razão E/E′ para o fluxo mitral (p = 0,02) e FEVE (p = 0,01). Adicionalmente, esses pacientes apresentavam níveis elevados de TNF-alfa (p = 0,05) e NT-proBNP (p = 0,03).

**Tabela 3:** – Comparação de variáveis ecocardiográficas e de biomarcadores em pacientes com DPOC em relação a ViAE abaixo ou acima de 36,5 mL/m **2**

Variáveis	ViAE > 36,5 ml/m^**2**^	ViAE ≤ 36,5 ml/m^**2**^	Valor p
E/E′ mitral	12,2 ± 4,5	8,0 ± 2,9	0,025
FEVE, %	51,5 ± 5,0	68,0 ± 4,1	0,012
TNF alfa	12,5 [10,1 a 22,3]	7,90 [6,7 a 11,5]	0,050
NT-proBNP	779,8 ± 322,3	271,0 ± 216,4	0,034

Valores apresentados como média ± desvio-padrão para variáveis com distribuição normal ou mediana e intervalo interquartil para variáveis não paramétricas. Comparação baseada no teste t ou Mann-Whitney para média e mediana, respectivamente; nível de significância p < 0,05. DPOC: doença pulmonar obstrutiva crônica; E/E′: velocidade de pico da onda E mitral na fase de enchimento rápido/velocidade de deslocamento do anel mitral septal ou lateral na fase de enchimento rápido; FEVE: fração de ejeção do ventrículo esquerdo; NT-proBNP: fragmento N-terminal do peptídeo natriurético tipo B; TNF: fator de necrose tumoral; ViAE: volume indexado do átrio esquerdo.

A Figura Central resume os principais resultados do presente estudo.

## Discussão

Os principais achados do presente estudo indicaram que, no momento da inclusão, os pacientes apresentaram aumento da média do ViAE, pressão sistólica da artéria pulmonar estimada elevada e níveis elevados de NT-proBNP. Aqueles que apresentaram desfechos desfavoráveis demonstraram piora dos sintomas e comprometimento da função pulmonar. O ViAE demonstrou boa acurácia na predição de hospitalização por exacerbação, e valores superiores a 36,5 mL/m^2^ foram associados a comprometimento da função diastólica e sistólica, bem como a níveis elevados de TNF-alfa e NT-proBNP.

Está bem estabelecido que pacientes com DPOC podem enfrentar aumento no risco de doenças cardiovasculares e mortalidade, independentemente dos fatores de risco conhecidos. Permanecem incertas as causas subjacentes desse fenômeno, além dos fatores de risco tradicionais. Um amplo estudo populacional envolvendo 5,8 milhões de indivíduos com mais de 40 anos de idade, dos quais 150.000 foram diagnosticados com DPOC, revelou que as taxas de infarto agudo do miocárdio, acidente vascular cerebral ou mortalidade cardiovascular nos 8 anos subsequentes foram 3,3 vezes maiores em pacientes com DPOC em comparação àqueles sem a doença.^17^ Além disso, outros estudos sugeriram um aumento de 20% a 30% nos eventos cardiovasculares e na mortalidade para cada redução de 10% no VEF_1_. Notavelmente, o perfil de pacientes com DPOC frequentemente se sobrepõe ao de pacientes com doença cardiovascular, incluindo maior prevalência do sexo masculino, idade avançada, histórico de tabagismo, sobrepeso e resistência à insulina.^18^

Dadas essas considerações, variáveis ecocardiográficas podem desempenhar um papel significativo na predição da gravidade da DPOC.^19^ Numerosos estudos focaram nos impactos da função do VE em pacientes com DPOC. Por exemplo, Anderson et al. relataram uma prevalência de 21,4% de hipertrofia do VE em homens e de 43,2% em mulheres com DPOC, independentemente da hipertensão. Esses achados sugerem que a DPOC tem um efeito independente na hipertrofia do VE.^20^

Ademais, vários estudos descreveram uma alta frequência de disfunção diastólica do VE em pacientes com DPOC em comparação com controles pareados por idade.^21^ Caram et al. identificaram uma associação significativa entre disfunção diastólica do VE e gravidade da DPOC.^11^ Mocan et al. relacionaram estados pró-inflamatórios sistêmicos na DPOC, caracterizados por níveis elevados de IL-6 e PCR, à disfunção diastólica do VE, concluindo que essas alterações estavam relacionadas à interdependência das variáveis e à sobrecarga das câmaras direitas frequentemente observada em pacientes com DPOC.^22^ Além disso, Kellerer et al., em 2021, constataram que a terapia de manutenção para DPOC, que melhorou a função pulmonar, estava associada ao tamanho do átrio esquerdo, reforçando o impacto da estabilização da DPOC na saúde cardiovascular.^23^

Até onde sabemos, este é o primeiro estudo a demonstrar o papel do ViAE como um preditor confiável de desfechos desfavoráveis, como hospitalização por exacerbação, em pacientes com DPOC. Além disso, o ViAE correlacionou-se com biomarcadores de desfechos desfavoráveis, incluindo TNF-alfa e NT-proBNP, destacando o estado pró-inflamatório da doença e sua correlação com condições cardiovasculares.

Apesar desses achados notáveis, é fundamental reconhecer as limitações do presente estudo, incluindo o pequeno tamanho da amostra e os desafios no recrutamento de pacientes devido à pandemia de COVID-19. Além disso, o presente estudo não considerou a divisão dos pacientes por grau de disfunção diastólica. A deformação atrial esquerda também não foi obtida para melhor avaliar a função diastólica e seu possível papel na fisiopatologia dos desfechos desfavoráveis em pacientes com ViAE aumentado.

A importância clínica do presente estudo reside na identificação de preditores para desfechos desfavoráveis em pacientes com DPOC, o que pode subsidiar o desenvolvimento de estratégias terapêuticas individualizadas. Esse conhecimento pode aprimorar os cuidados cardiovasculares e respiratórios, apoiar práticas de reabilitação que visam melhorar a tolerância ao exercício e a qualidade de vida e potencialmente alterar o curso da doença. Estudos futuros são necessários para explorar e incorporar novas estratégias de avaliação no acompanhamento de pacientes com DPOC.

Finalmente, a identificação de índices prognósticos, como o ViAE e outros marcadores explorados no presente estudo, pode constituir escores potenciais para orientar o tratamento farmacológico em pacientes com DPOC que apresentam doença cardiovascular. Ao direcionar intervenções farmacológicas personalizadas (como agentes anti-inflamatórios e broncodilatadores) para aqueles com maior risco, os profissionais de saúde podem controlar os sintomas de forma mais eficaz, reduzir a incidência de exacerbações e melhorar a saúde cardiovascular geral. Essa abordagem individualizada não apenas melhora os resultados dos pacientes, mas também otimiza a alocação de recursos em ambientes clínicos.

## Conclusão

O ViAE pode servir como um marcador confiável para predizer desfechos desfavoráveis em pacientes com DPOC. Sua associação com níveis elevados de TNF-alfa e NT-proBNP reforça o estado pró-inflamatório da DPOC e seu impacto no sistema cardiovascular. Esses achados destacam a necessidade de estratégias individualizadas destinadas a melhorar a gravidade da doença e a função cardiovascular nessa população de pacientes.
