# Supplementary material for: O Papel do Volume Indexado do Átrio Esquerdo na Detecção de Desfechos Desfavoráveis em Pacientes com Doença Pulmonar Obstrutiva Crônica: Estudo de Coorte
Source: Arq Bras Cardiol. 2025 Nov 19;122(10):e20250131. [Article in Portuguese] doi: 10.36660/abc.20250131 (PMC12978166; doi:10.36660/abc.20250131)
Supplement: Translation

# Translation: The Role of Indexed Left Atrial Volume in Detecting Adverse Outcomes in Patients with Chronic Obstructive Pulmonary Disease: A Cohort Study

**DOI:** 10.36660/abc.20250131i

**Authors:** Maria Clara C Sposito, Leticia A. Branco, Jaqueline Bianchi, Fernanda O. Duarte, Krissia F. Godoy, Joice M. A. Rodolpho, Bruna Dias de Lima Fragelli, Renan S. Marinho, Stella Maris Firmino, Audrey Borghi Silva, Fernanda Freitas Anibal, Meliza Goi Roscani

**Affiliations:** 1 Brazil Universidade Federal de São Carlos, São Carlos, SP – Brazil

**Keywords:** Pulmonary Emphysema, Prognosis, Biomarkers

## Abstract

**Background:**

Chronic obstructive pulmonary disease (COPD) is a progressive disease with high mortality, and understanding the predictors of adverse outcomes can aid in developing individualized therapeutic approaches and in making decisions regarding hospital or intensive care unit admissions.

**Objective:**

To assess the epidemiological and laboratory profiles of patients with COPD and to correlate clinical, echocardiographic, and biomarker variables with unfavorable outcomes due to exacerbation.

**Method:**

This was a prospective study involving patients with COPD who underwent clinical evaluation, spirometry, transthoracic echocardiography, and laboratory tests. Patients were followed for 1 year to monitor for unfavorable outcomes. The significance level considered for all analyses was p < 0.05.

**Results:**

A total of 228 patients were included, with the mean age of 71 ± 9 years. The majority of patients were male (58%), and smoking (72%), hypertension (66%), and GOLD B (55%) were predominant. Indexed left atrial volume (ILAV) demonstrated good accuracy in detecting hospitalization for exacerbation, with a cutoff point of 36.5 ml/m^2^, yielding 100% sensitivity and 70% specificity. Patients with ILAV > 36.5 ml/m^2^exhibited worsening diastolic and systolic function, as indicated by mitral E wave peak velocity in the rapid filling phase/E′ velocity of displacement of the septal or lateral mitral annulus in the rapid filling phase (E/E′) (p = 0.02) and left ventricle ejection fraction (p = 0.01), along with elevated levels of tumor necrosis factor alpha (p = 0.05) and NT-proBNP (p = 0.03).

**Conclusion:**

ILAV may be a reliable marker for predicting unfavorable outcomes in patients with COPD. Individualized strategies should be implemented to improve disease severity and cardiovascular function.

## Introduction

Chronic obstructive pulmonary disease (COPD) is a progressive condition associated with high mortality and morbidity, representing a significant public health challenge. Well-established prognostic markers, such as age, forced expiratory volume in one second (FEV_1_), body mass index, and smoking status can predict adverse outcomes, including death, risk of acute exacerbation, and hospital readmission in patients with COPD.^1 , 2^Studies have indicated pro-adrenomedullin and the 6-minute walk test as independent predictors of 2-year mortality in COPD patients. Additionally, other studies have highlighted the importance of biomarkers such as D-dimer, troponin, B-type natriuretic peptide (BNP), interleukin 6 (IL-6), interleukin 8 (IL-8) and C-reactive protein (CRP) as significant predictors of adverse outcomes in COPD.^3^

The coexistence of heart failure in patients with COPD is well recognized, as these conditions share several common risk factors, including smoking, systemic inflammation, and advanced age. Research suggests that elevated levels of CRP and BNP, secreted by damaged cardiomyocytes, are prevalent in COPD. Furthermore, studies have underscored the significance of D-dimer, troponin, IL-6 and IL-8 as important predictors of adverse outcomes in these patients.^4 - 6^Troponin, as noted in several studies, may be elevated in patients hospitalized due to COPD exacerbations.^7^

Given the association of cardiovascular comorbidities in patients with COPD, it is believed that the echocardiography may play a crucial role in detecting signs of disease severity.^8^Cassagnes et al. found that indexed left atrial volume (ILAV) was a marker of unfavorable progression in patients with COPD. However, another study reported no difference in in left atrial strain between patients with COPD and healthy individuals.^9^The relationship between echocardiographic variables and prognostic biomarkers in predicting adverse outcomes in COPD remains inadequately elucidated.^10^Rawy et al. observed a high prevalence of left ventricular (LV) diastolic dysfunction in patients with COPD, which was associated with increased disease severity and elevated levels of inflammatory markers, independent of other biomarkers, such as CRP levels.^10 , 11^

Considering these findings, the quantification, recording, monitoring, and analysis of these variables and their associations may play an important role in defining prognosis and identifying patients with greater severity and unfavorable disease progression. Moreover, early detection of prognostic markers for coexisting cardiovascular conditions could facilitate earlier pharmacological strategies, potentially altering the disease course and improving patient survival.

Therefore, the aim of this study was to correlate clinical, echocardiographic, and biomarker data with adverse outcomes in patients with COPD and to determine whether the association of echocardiographic and laboratorial parameters can better discriminate disease severity.

## Methods

A cohort study was conducted to follow patients with a confirmed diagnosis of COPD attending a pulmonary outpatient clinic. The project received approval from the National Health Research Committee and the Research Ethics Committees of the Health Centers (CAAE 63414822.1.0000.5504).

This study was performed from January 2022 to October 2023 and adhered to the STROBE (STrengthening the Reporting of OBservational studies in Epidemiology) guidelines.

Patient selection was based on the assessment of electronic records from outpatient consultations.

Inclusion criteria were: informed consent obtained; age over 40 years, confirmed diagnosis of COPD, defined as post-bronchodilator spirometry showing FEV_1_/vital forced capacity (FVC) < 70%; and absence of physical limitations.

Exclusion criteria were: patients with biomechanical or psychiatric limitations that hindered their ability to complete questionnaires or undergo examinations; and patients experiencing an exacerbation of COPD in the 3 months prior to inclusion.

At the time of inclusion, all patients underwent clinical evaluation, a transthoracic echocardiogram performed by a cardiologist with extensive experience, and a pre- and post-bronchodilator spirometry conducted by a trained pulmonologist. Additionally, blood laboratory tests were collected. All included subjects were monitored for 1 year to investigate primary endpoints, which included hospitalization due to disease exacerbation (characterized by increased dyspnea and/or cough and sputum worsening within 14 days, accompanied by tachypnea or tachycardia) and mortality.^12^Data on primary endpoints were obtained by electronic records and follow-up phone calls to patients or their family members.

### Clinical evaluation

Patients were assessed for symptoms such as cough, sputum production, sputum color, shortness of breath, exercise tolerance, prior hospitalization due to exacerbation, smoking status, use of home oxygen therapy, current medications, and presence of comorbidities including systemic arterial hypertension, diabetes mellitus, heart failure, coronary artery disease, chronic kidney disease, and obesity. The modified Medical Research Council (mMRC) dyspnea scale and the COPD assessment test (CAT) were also employed during clinical assessments. A comprehensive physical examination included measurements of systemic arterial pressure, respiratory rate, heart rate, oxygen saturation, body mass index, and presence of pulmonary rales.^13 , 14^

### Doppler echocardiography

For clinical and diagnostic stratification, patients with COPD underwent a 2D-echocardiogram using an “Affiniti 50” ultrasound device (Philips, USA) with a 2–4 MHz transducer and tissue Doppler imaging software. A single cardiologist performed all assessments. Quantification of cardiac chambers was conducted according to the American Society of Echocardiography guidelines.^15^LV diastolic diameter, ILAV (via Simpson method), indexed LV mass, relative wall thickness, and basal right ventricular diastolic diameter were measured. Right ventricular dimensions were estimated following recent guidelines, utilizing a right ventricular-focused apical 4-chamber view. Right ventricular diastolic diameter was defined as the maximum transverse dimension in the basal third of the right ventricular inflow tract at end-diastole. Mitral and tricuspid inflow patterns were assessed, measuring early (E) and late (A) diastolic filling velocities, and calculating the E/A ratio. Longitudinal velocities of both the left and right ventricles at the respective mitral and tricuspid annular levels were obtained using color-guided pulsed-wave tissue Doppler in apical 4-chamber view. Spectral Doppler measurements included peak systolic longitudinal velocity (s′) and early diastolic filling velocity (e′), averaged over 5 consecutive cardiac cycles. The E/e′ ratio was calculated to assess diastolic function, while left ventricular ejection fraction (LVEF) was derived using the Teicholz method, contingent upon the absence of regional wall motion abnormalities. Right ventricular systolic function was evaluated through subjective wall motion analysis, tricuspid annular plane systolic excursion, and peak tricuspid annular systolic velocity.

### Spirometry

Pre- and post-bronchodilator spirometry were obtained (Masterscreen Body, Mijnhardt/Jäger, Würzburg, German) that provided measurements of the FEV_1_and FVC, allowing for calculation of the FEV_1_/FVC ratio. Spirometry was performed in accordance with the recommendations of the American Thoracic Society/European Respiratory Society guidelines.^16^The severity of airflow limitation in COPD was classified based on Global Initiative for Chronic Obstructive Lung Disease (GOLD) criteria, categorizing patients as having moderate (GOLD II), severe (GOLD III), or very severe (GOLD IV) disease.^12^

### Symptom assessment

Patients’ symptoms were evaluated at 12 months of follow-up using the mMRC dyspnea scale, which measures the severity of dyspnea experienced by the patient. Additionally, the CAT was utilized to assess impact of symptoms on patients with COPD; however, the CAT does not classify patients into severity categories. CAT scores greater than 25 are infrequent in healthy patients, while scores below 25 are uncommon in patients with COPD.^13 , 14^

### Biomarkers

The following blood laboratory tests were collected at inclusion: D-dimer, NT-proBNP, cortisol, CRP, high-sensitivity CRP, IL-6, IL-8, and tumor necrosis factor (TNF) alpha.

### Follow-up procedures

Patients were included over a 12-month period by telephone calls and routine medical consultation with their family caregivers. Investigations focused on unfavorable outcomes, with medical records reviewed during both the telephone calls and consultations.

### Statistical analysis

All analyses were performed in Sigma Plot Systat, version 12.0. Results were reported as mean and standard deviation for continuous variables with normal distribution, as median and interquartile range for continuous variables without normal distribution, and as frequencies and percentage for categorical variables. The non-paired Student’s t test was applied for comparisons of continuous variables, while the Mann Whitney U test was utilized for ordinal variables. The Shapiro-Wilk test was performed to check the normality of the data. The chi-squared test was employed to assess independence for categorical variables. Multiple linear regression was performed. A multiple linear regression analysis was performed to predict the values of dependent variables. Finally, receiver operating characteristic curves were constructed for continuous and ordinal variables concerning outcomes such as death, exacerbation, hospitalization, emergency room visits, and negative outcomes, with the area under the curve (AUC) calculated to assess diagnostic performance. The significance value considered for all the analyses was p < 0.05.

## Results

A total of 228 patients were included in the study. The baseline clinical and laboratory characteristics are displayed in Table 1 . The mean age was 71 ± 9 years, with notable prevalence of male sex and systemic arterial hypertension. The majority of patients were classified as GOLD group B. Primary endpoints were observed in approximately 7.9% of the cohort. Echocardiographic assessment revealed preserved LVEF of 63.9% ± 7.5%. However, there was an increase in the mean ILAV of 42.7 ± 28.2 ml/m^2^, along with an estimated pulmonary artery systolic pressure of 46.8 ± 15.4 mmHg. Additionally, NT-proBNP levels were elevated at the time of inclusion.

**Table 1:** – Baseline clinical and laboratory characteristics of patients with COPD

Variables	N = 228
Age, years	71 ± 9
Male sex, n (%)	132 (57.9)
Active smoker, n (%)	62 (27.1)
Former smoker, n (%)	150 (65.8)
Smoking time, years	37.2 ± 18.3
Smoking history, pack-years	63.2 ± 55.2
Time without smoking, years	10.3 ± 12.7
**Comorbidities**	
Asthma, n (%)	30 (13.2)
Diabetes mellitus type 2, n (%)	32 (14.0)
SAH, n (%)	151 (66.3)
CAD, n (%)	4 (1.8)
Obesity, n (%)	30 (13.1)
BMI (kg/m^2^)	25.6 ± 8.0
**Symptom severity score**	
mMRC (0 to 5)	3.0 [1.0 a 3.0]
CAT score (0 to 40)	19.0 [9.0 a 25.0]
**Spirometry**	
FVC, L	2.5 ± 1.0
FVC, %	74.2 ± 24.1
FEV_1_, L	1.5 ± 0.7
FEV_1_, %	56.1 ± 21.4
FEV_1_/FVC	0.6 ± 0.2
FEV_1_/FVC, %	18.3 ± 37.5
FEV_1_/FVC post-bronchodilator, L	0.7 ± 0.1
FEV_1_/FVC post-bronchodilator, %	50.0 ± 48.3
**GOLD and ABE classification**	
**GOLD**	2.0 [2.0 a 3.0]
**ABE Classification**	
A	24.0%
B	56.0%
E	20.0%
**Echocardiogram**	
LVDD, mm	47.7 ± 10.0
Basal RVDD, mm	33.9 ± 9.4
Relative wall thickness	0.4 ± 0.1
ILAV, ml/m^2^	39.92 ± 19.33
ILVM, g/m^2^	99.6 ± 33.3
IRAV, ml/m^2^	38.3 ± 46.2
Mitral E, cm/s	66.5 ± 20.8
Mitral S′, cm/s	9.1 ± 2.2
Mitral septal annulus E′, cm/s	7.5 ± 2.7
Mitral lateral annulus E′, cm/s	8.8 ± 3.1
Mitral E/E′	10.2 ± 4.0
Tricuspid E, cm/s	44.0 ± 13.1
Pulmonary artery systolic pressure, mmHg	46.8 ± 15.4
TAPSE, mm	20.9 ± 6.1
LVEF, %	63.9 ± 7.5
LV global longitudinal strain, %	−12.2 ± 14.5
**Biomarkers**	
D-dimer, mg/L	0.7 ± 0.8
Myoglobin, ng/mL	17.6 ± 14.8
NT-proBNP, pg/ mL	330.0 [234.2 a 599.0]
CRP, mg/L	6.8 ± 6.9
Hs-CRP, mg/L	4.6 ± 6.6
IL-1, pg/mL	6.8 ± 5.8
IL-6, pg/mL	6.9 ± 5.4
IL-8, pg/mL	13.2 ± 10.4
TNF-alpha, pg/mL	15.0 ± 22.9
**Medications, n (%)**	
SABA	96 (42.1)
LABA + IC	54 (23.4)
LAMA	44 (19.3)
LABA + LAMA	3 (1.3)
Primary endpoints, n (%)	18 (7.9)
Death	4 (1.8)
Hospitalization due to exacerbation	14 (6.1)

Values presented as mean ± standard deviation for variables with normal distribution or median and interquartile range for non-parametric variables or number (N) and percentage (%). Reference values used – D-dimer: 0 to 0.5 mg/L; CK-MB: 0 to 5 ng/ml; NT-proBNP: 0 to 300 pg/mL < 75 years and 0 to 450 ≥ 75 years; CRP: 0 to 10 mg/L; Hs-CRP: 0 to 1 mg/L; myoglobin: 0 to 58 ng/mL; IL-6: < 5.9 pg/ML; IL-1: < 5 pg/mL; TNF-alpha: < 8.1 pg/mL; IL-8: < 62 pg/mL. GOLD ABE classification – A: non-exacerbators, symptoms low; B: non-exacerbators, symptoms high; E: exacerbators. BMI: body mass index; CAD: coronary artery disease; CAT: COPD assessment test; CRP: C-reactive protein; E: E wave peak velocity in the mitral or tricuspid rapid filling phase; FEV1: forced expiratory volume at the first second; FVC: forced vital capacity; GOLD: Global Initiative for Chronic Obstructive Lung Disease; Hs-CRP: high-sensitivity C-reactive protein; IC: inhaled corticosteroids; ILAV: indexed left atrial volume; ILVM: indexed left ventricular mass; IRAV: indexed right atrial volume; LABA: long-acting beta agonist; LAMA: long-acting muscarinic antagonist; LVDD: left ventricular diastolic diameter; LVEF: left ventricular ejection fraction; mitral E′: velocity of displacement of the septal or lateral mitral annulus in the rapid filling phase; mMRC: modified Medical Research Council dyspnea scale; NT-proBNP: N-terminal pro-B-type natriuretic peptide; RVDD: right ventricular diastolic diameter; SABA: short-acting beta agonist; SAMA: short-acting muscarinic antagonist; TAPSE: tricuspid annular plane systolic excursion; TNF: tumor necrosis factor; tricuspid E′: velocity of displacement of the lateral tricuspid annulus in the rapid filling phase.

Patients who experienced unfavorable outcomes, including death and/or hospitalization due to exacerbation, exhibited worse symptoms and more impaired pulmonary function at the time of inclusion, as shown in Table 2 .

**Table 2:** – Comparison of patients with COPD in relation to primary endpoints over a 1-year follow-up period

Variable	Primary endpoints, yes (N = 18)	Primary endpoints, no (N = 210)	p value
CAT score	26 [15 a 31]	17 [7 a 20]	**0.032**
Limitation in activities (0 to 5)	5.0 [3.3 a 5.0]	0.0 [0.0 a 3.0]	**0.019**
Confident to leave the house (0 to 5)	1.0 [0.0 a 5.0]	0.0 [0.0 a 0.0]	**0.041**
GOLD	3.0 [2.0 a 3.5]	2.0 [2.0 a 3.0]	**0.039**
FVC, %	53.3 ± 14.7	69.2 ± 18.2	**0.02**
FEV1, %	43.2 ± 17.4	59.0 ± 17.7	**0.025**
FEV1, post-bronchodilator %	46.1 ± 17.7	60.4 ± 16.8	0.063

Values presented as mean ± standard deviation for normally distributed variables or median and interquartile range for non-parametric variables. Comparison based on t test or Mann-Whitney for mean and median, respectively; significance level p < 0.05. COPD: chronic obstructive pulmonary disease; FEV1: forced expiratory volume in the first second; FVC: forced vital capacity.

In an isolated analysis, patients who required hospitalized for exacerbation over the 1-year period showed a significant increase in ILAV (p = 0.008), as shown in the Figure 1 . In a multiple linear regression adjusted for CAT score, only NT-proBNP (p = 0.03) and CRP (p = 0.03) were found to be significantly associated with the increased ILAV (R = 0.83; p = 0.006).

**Figure 1:** – Comparison of ILAV (mL/m2) between patients who experienced hospitalization for exacerbation of COPD and those who did not. COPD: chronic obstructive pulmonary disease. ILAV: indexed left atrial volume.

Considering the clinical and ultrasonographic variables, ILAV demonstrated good accuracy in predicting hospitalization due to exacerbation. The ROC curve indicated an AUC of 0.90 (95% confidence interval: 0.70 to 1.10; p = 0.04) with a cutoff point of > 36.5 ml/m^2^. This threshold exhibited a sensitivity of 100% and a specificity of 70% for predicting this adverse outcome. These results are presented in Figure 2 .

**Figure 2:** *–* ROC curve analysis shows an AUC of 0.90 (95% confidence interval: 0.70 to 1.10; p = 0.04), with a sensitivity of 100% and a specificity of 70%. An optimal ILAV cutoff of > 36.5 mL/m2 was identified for predicting hospitalization due to exacerbations in patients with COPD during the 1-year follow-up period. AUC: area under the curve; COPD: chronic obstructive pulmonary disease; ILAV: indexed left atrial volume; ROC: receiver operating characteristic.

Using the cutoff point identified in the ROC analysis, we compared the variables for ILAV values below or greater than 36.5 ml/m^2^, as presented in Table 3 . Patients with ILAV > 36.5 ml/m^2^exhibited worsening diastolic and systolic function, indicated by E/E′ ratio for mitral inflow (p = 0.02) and LVEF (p=0.01). Additionally, these patients had elevated levels of TNF-alpha (p = 0.05) and NT-proBNP (p = 0.03).

**Table 3:** – Comparison of echocardiographic and biomarker variables in patients with COPD in relation to ILAV below or above 36,5 mL/m **2**

Variables	ILAV > 36,5 ml/m^**2**^	ILAV ≤ 36,5 ml/m^**2**^	p value
Mitral E/E′	12.2 ± 4.5	8.0 ± 2.9	0.025
LVEF, %	51.5 ± 5.0	68.0 ± 4.1	0.012
TNF-alpha	12.5 [10.1 a 22.3]	7.90 [6.7 a 11.5]	0.050
NT-proBNP	779.8 ± 322.3	271.0 ± 216.4	0.034

Values presented as mean ± standard deviation for normally distributed variables or median and interquartile range for non-parametric variables. Comparison based on t test or Mann-Whitney for mean and median, respectively; significance level p < 0.05. COPD: chronic obstructive pulmonary disease; E/E′: E wave peak velocity in the mitral rapid filling phase/velocity of displacement of the medium of septal or lateral mitral annulus in the rapid filling phase; ILAV: indexed left atrial volume; LVEF: left ventricular ejection fraction; NT-proBNP: N-terminal pro-B-type natriuretic peptide; TNF: tumor necrosis factor.

The Central Illustration summarizes the main results of the present study.

## Discussion

The main findings of this study indicated that, at the time of inclusion, patients exhibited increased mean of ILAV, elevated estimated pulmonary artery pressure, and high levels of NT-proBNP. Those experiencing adverse outcomes demonstrated worsening symptoms and impaired pulmonary function. Notably, ILAV showed good accuracy in predicting hospitalization for exacerbation, and values exceeding 36.5 mL/m^2^were associated with impaired diastolic and systolic function, as well as elevated levels of TNF-alpha and NT-proBNP.

It is well established that patients with COPD may face increased cardiovascular disease risk and mortality, independent of known risk factors. The underlying causes of this phenomenon, beyond traditional risk factors, remain uncertain. A large population study involving 5.8 million individuals over 40 years of age, of whom 150,000 were diagnosed with COPD, revealed that the rates of acute myocardial infarction, stroke, or cardiovascular mortality over the following 8 years were 3.3 times higher in patients with COPD compared to those without the disease.^17^Additionally, other studies have suggested a 20% to 30% increase in cardiovascular events and mortality for each 10% decline in FEV_1_. Notably, the profile of patients with COPD often overlaps with that of patients with cardiovascular disease, including a higher prevalence of male sex, advanced age, smoking history, overweight status, and insulin resistance.^18^

Given these considerations, echocardiographic variables may play significant role in predicting the severity of COPD.^19^Numerous studies have focused on the impacts of LV function in patients with COPD. For instance, Anderson et al. reported a 21.4% prevalence of LV hypertrophy in men and a 43.2% prevalence in women with COPD, independent of hypertension. These findings suggest that COPD has an independent effect on LV hypertrophy.^20^

Furthermore, several studies have described a high frequency of LV diastolic dysfunction in patients with COPD compared to age-matched controls.^21^Caram et al. identified a significant association between LV diastolic dysfunction and severity of COPD.^11^Mocan et al. linked systemic pro-inflammatory states in COPD, characterized by elevated levels of IL-6 and CRP, to LV diastolic dysfunction, concluding that these changes were related to the interdependence of the variables and right chamber pressure overload commonly observed COPD patients.^22^Additionally, Kellerer et al. in 2021 found that maintenance therapy for COPD, which improved lung function, was associated with left atrial size, reinforcing the impact of COPD stabilization on cardiovascular health.^23^

To the best of our knowledge, this study is the first to demonstrate the role of ILAV as a reliable predictor of adverse outcomes, such as hospitalization for exacerbation, in patients with COPD. Moreover, ILAV correlated with biomarkers of unfavorable outcomes, including TNF-alpha and NT-proBNP, highlighting the pro-inflammatory state of the disease and its correlation with cardiovascular conditions.

Despite these noteworthy findings, it is essential to acknowledge the limitations of this study, including the small sample size and challenges in patient recruitment due to the COVID-19 pandemic. Additionally, this study did not consider the division of patients by degree of diastolic dysfunction. Left atrial strain was also not performed to better assess diastolic function and its possible role in the pathophysiology of unfavorable outcomes in patients with increased ILAV.

The clinical importance of the present study lies in the identification of predictors for unfavorable outcomes in patients with COPD, which could inform the development of individualized therapeutic strategies. This knowledge can enhance cardiovascular and respiratory care, support rehabilitation practices aimed at improving exercise tolerance and quality of life, and potentially alter the disease trajectory. Future studies are warranted to further explore and incorporate new strategies for assessment in the follow-up of patients with COPD.

Finally, the identification of prognostic indices, such as ILAV and other markers explored in the present study, may constitute potential scores for guiding pharmacological treatment in COPD patients that present cardiovascular disease. By targeting those at higher risk with tailored pharmacological interventions (such as anti-inflammatory agents and bronchodilators) healthcare providers can more effectively manage symptoms, reduce the incidence of exacerbations, and improve overall cardiovascular health. This individualized approach not only enhances patient outcomes but also optimizes resource allocation in clinical settings.

## Conclusion

ILAV may serve as a reliable marker for predicting unfavorable outcomes in patients with COPD. Its association with elevated levels of TNF-alpha and NT-proBNP reinforces the pro-inflammatory state of COPD and its impact on the cardiovascular system. These findings highlight the need for individualized strategies aimed at improving both disease severity and cardiovascular function in this patient population.
